# Partial Exsolution Enables Superior Bifunctionality of Ir@SrIrO_3_ for Acidic Overall Water Splitting

**DOI:** 10.1002/advs.202309750

**Published:** 2024-04-02

**Authors:** Ling Zhao, Zetian Tao, Maosheng You, Huangwei Xiao, Sijiao Wang, Wenjia Ma, Yonglong Huang, Beibei He, Qi Chen

**Affiliations:** ^1^ School of Marine Science and Engineering Hainan University Haikou 570228 P. R China; ^2^ Faculty of Materials Science and Chemistry China University of Geosciences Wuhan 430074 P. R. China; ^3^ School of Resources, Environment and Safety Engineering University of South China Hengyang Hunan 421001 P. R. China

**Keywords:** acidic media, bifunctional, perovskite, partial exsolution, water splitting

## Abstract

The pursuit of efficient and durable bifunctional electrocatalysts for overall water splitting in acidic media is highly desirable, albeit challenging. SrIrO_3_ based perovskites are electrochemically active for oxygen evolution reaction (OER), however, their inert activities toward hydrogen evolution reaction (HER) severely restrict the practical implementation in overall water splitting. Herein, an Ir@SrIrO_3_ heterojunction is newly developed by a partial exsolution approach, ensuring strong metal‐support interaction for OER and HER. Notably, the Ir@SrIrO_3_‐175 electrocatalyst, prepared by annealing SrIrO_3_ in 5% H_2_ atmosphere at 175 °C, delivers ultralow overpotentials of 229 mV at 10 mA cm^−2^ for OER and 28 mV at 10 mA cm^−2^ for HER, surpassing most recently reported bifunctional electrocatalysts. Moreover, the water electrolyzer using the Ir@SrIrO_3_‐175 bifunctional electrocatalyst demonstrates the potential application prospect with high electrochemical performance and excellent durability in acidic environment. Theoretical calculations unveil that constructing Ir@SrIrO_3_ heterojunction regulates interfacial electronic redistribution, ultimately enabling low energy barriers for both OER and HER.

## Graphical abstract

An Ir@SrIrO_3_ heterojunction with strong metal‐support interaction is prepared by a top‐down partial exsolution strategy for acidic overall water splitting. Thanks to interfacial electronic redistribution and reduced energy barriers for oxygen evolution reaction and hydrogen evolution reaction, this advanced electrocatalyst achieves high bifunctional activity and good durability, showing great promise for commercial application.

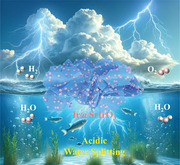

## 1 Introduction

Hydrogen, being an increasingly prominent energy carrier, has garnered substantial attention because of its environmentally friendly and sustainable natures. Electrochemical water splitting, particularly driven by renewable solar energy and wind power, is widely considered as a promising technology for hydrogen production.^[^ ^1^ ^]^ In contrast to industrially mature alkaline water electrolysis,^[^ ^2^ ^]^ acidic water splitting^[^ ^3^ ^]^ utilizing proton exchange membrane (PEM) technology boasts high energy efficiency, efficient gas separation and impressive tolerance to environmental impurities, making it crucial for the scalable application of hydrogen generation. Acidic water splitting involves two essential half‐electrochemical reactions, including anodic oxygen evolution reaction (OER) and cathodic hydrogen evolution reaction (HER). Nevertheless, a grand challenge in the acidic water splitting lies in the sluggish reaction kinetics, especially for the complex four‐electron OER process.^[^ ^4^ ^]^ Moreover, the highly corrosive acidic environment readily causes the dissolution and degradation of electrocatalysts,^[^ ^5^ ^]^ ultimately diminishing the energy efficiency of overall water splitting. Up to this point, noble metal based IrO_2_ (or RuO_2_)^[^ ^6^ ^]^ and Pt/C^[^ ^7^ ^]^ have been recognized as state‐of‐the‐art electrocatalysts towards OER and HER, respectively. Unfortunately, their limited availability, prohibitive cost, and inferior bifunctionality severely inhibit their commercial prospects.^[^ ^8^ ^]^ Therefore, to advance economical and design‐simplified acidic water splitting,^[^ ^9^ ^]^ developing bifunctional electrocatalysts capable of efficiently driving both OER and HER in acidic media is of great significance, yet remains a pressing challenge.

Multi‐metal perovskite oxides, including SrIrO_3_,^[^ ^10^ ^]^ SrRuO_3_,^[^ ^11^ ^]^ and their derivatives,^[^ ^12^ ^]^ have emerged as promising alternative electrocatalysts to IrO_2_ (or RuO_2_) toward acidic OER, since they essentially improve the atomic utilization of noble metals. These perovskite oxides possess the unique features of compositional variability and electronic structure flexibility. As revealed, the activity origin of SrIrO_3_ related‐perovskite oxides is primarily determined by local electron structure,^[^ ^13^ ^]^ IrO_6_ octahedron,^[^ ^10^, ^14^ ^]^ and surface reconstruction.^[^ ^15^ ^]^ Despite the significant successes in OER, the HER activity of pristine SrIrO_3_ electrocatalyst remains inadequate due to the fundamentally different HER mechanism in contrast to OER. On the other side, metallic Ir‐based materials offer favorable adsorption energy for HER intermediate, thereby demonstrating high catalytic activity towards HER rather than OER.^[^ ^16^ ^]^

Constructing heterojunction is a powerful strategy for realizing the multifunctionally in electrocatalysis, because the heterojunction not only inherits the merit of individual components, but also steers the synergistic effect among coupled components.^[^ ^17^ ^]^ In particular, Janus metal/semiconductor heterojunctions, such as Ru/RuS_2_^[^ ^18^ ^]^ and Ru/RuO_2_,^[^ ^19^ ^]^ have shown the ability to optimize the bifunctional activity. Benefiting from the Mott‐Schottky effect in the heterojunctions, the interfacial electronic structure can be regulated. However, engineering metal/perovskite heterojunction for acidic bifunctional OER and HER and probing the underlying interfacial interaction have rarely been reported.

Taking inspiration from the above view, we present the elaboration of a novel Ir@SrIrO_3_ heterojunction prepared via a partial exsolution strategy for acidic overall water splitting. Compared to the traditional bottom‐up synthetic approaches, this top‐down exsolution route ensures a strong metal‐support interaction between exsolved nanoparticles and host materials.^[^ ^20^ ^]^ As anticipated, the Ir@SrIrO_3_‐175 heterojunction displays significantly enhanced bifunctional activity, compared to the pristine SrIrO_3_ perovskite. The Ir@SrIrO_3_‐175 electrocatalyst demonstrates an ultralow overpotential of 229 mV for achieving a current density of 10 mA cm^−2^ toward OER. Furthermore, the HER activity of Ir@SrIrO_3_‐175 electrocatalyst is activated, requiring only a low overpotential of 28 mV at 10 mA cm^−2^. As a proof‐of‐concept, the assembled electrolyzer using the Ir@SrIrO_3_‐175 bifunctional electrocatalyst exhibits remarkable activity and long‐life durability. Theoretical calculations unveil that the metal‐perovskite interaction leads to the interfacial electronic redistribution in the Ir@SrIrO_3_ heterojunction, which reduces the energy barriers of OER and HER.

## 2 Results and Discussion

### 2.1 Morphology and Structure

**Figure 1:**
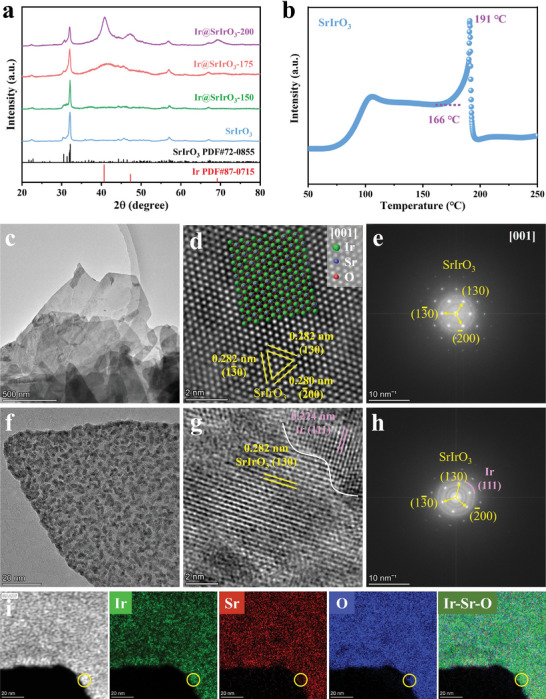
Structural and morphological characterizations of electrocatalysts. a) XRD patterns of SrIrO_3_, Ir@SrIrO_3_‐150, Ir@SrIrO_3_‐175, Ir@SrIrO_3_‐200, b) H_2_‐TPR curve of SrIrO_3_ under 5% H_2_‐95% Ar atmosphere from RT to 250 °C, c) Low magnification TEM image, d) High magnification TEM image, e) SAED image of pristine SrIrO_3_, f) Low magnification TEM image, g) High magnification TEM image, h) SAED image, and i) Elemental mapping of Ir@SrIrO_3_‐175.

The Ir@SrIrO_3_ heterojunction was synthesized through a top‐down partial exsolution strategy, as illustrated in Figure S1, Supporting Information. Briefly, the initial SrIrO_3_ nanosheets were prepared using a facile sol‐gel method,^[^ ^10b^ ^]^ followed by annealing in 5% H_2_ atmosphere at different temperatures (150 °C, 175 °C, and 200 °C) to receive exsolved heterojunctions (denoted as Ir@SrIrO_3_‐150, Ir@SrIrO_3_‐175, and Ir@SrIrO_3_‐200). During exsolution process, Ir cations in perovskite lattices were partially reduced to metallic state and were segregated onto the SrIrO_3_ surface, yielding Ir@SrIrO_3_ heterojunctions. It is well accepted that those exsolved metal nanoparticles are closely embedded into the perovskite matrixes, ensuring a strong metal‐support interaction.^[^ ^21^ ^]^ As depicted in X‐ray diffraction (XRD) patterns (**Figure** **1**a), the as‐prepared SrIrO_3_ adopts a monoclinic perovskite structure, well indexed to the diffraction peaks of JCPDS‐72–0855.^[^ ^13b^ ^]^ Upon annealing at (or above) 175 °C, cubic metallic Ir is generated.^[^ ^22^ ^]^ Rietveld refinements were performed to identify the crystallography of Ir@SrIrO_3_‐150, Ir@SrIrO_3_‐175, and Ir@SrIrO_3_‐200 (Figure S2 and Table S1, Supporting Information). The results further demonstrates that both Ir@SrIrO_3_‐175 and Ir@SrIrO_3_‐200 electrocatalysts consist of the SrIrO_3_ perovskite phase with a space group of C2/c and the metallic Ir phase with a space group of Fm‐3m. H_2_ temperature‐programmed reduction (H_2_‐TPR) analysis further confirms that the hydrogen consumption peak is beginning around 166 °C (Figure 1b),^[^ ^23^ ^]^ which aligns well with the exsolution behavior of SrIrO_3_ from XRD results.

As observed by scanning electron microscopy (SEM), the as‐synthesized SrIrO_3_ displays the morphology of nanosheets, which almost remains intact after exsolution process (Figure S3, Supporting Information). These nanosheets promise abundant exposed active sites for electrocatalytic reactions.^[^ ^24^ ^]^ Particularly, the microstructures of pristine SrIrO_3_ (Figure 1c–e) and Ir@SrIrO_3_‐175 heterojunction (Figure 1f–i) were further investigated via transmission electron microscopy (TEM). For the pristine SrIrO_3_, the high‐angle annular dark‐field scanning TEM (HAADF‐STEM) displays that the precise atomic arrangement agrees well with the monoclinic SrIrO_3_ perovskite along the [001] zone axis (Figure 1d). Thus, the exposed plane of SrIrO_3_ nanosheets is the (001) facet. In the selected area electron diffraction (SAED) image, three sets of lattice fringes with the interplanar spacings of 0.282 nm, 0.282 nm, and 0.280 nm agree well with (1 3 0), (1 –3 0), and (‐2 0 0) crystal faces of SrIrO_3_, respectively (Figure 1e).

As a contrast, the Ir@SrIrO_3_ heterojunction exhibits a coarser surface following the exsolution treatment (Figure 1f). Distinct interplanar distances of 0.224 nm and 0.282 nm are in accordance with the (1 1 1) lattice plane of metallic Ir and the (1 3 0) lattice plane of SrIrO_3_, respectively (Figure 1g). Results from SAED image further confirm the coexistence of metallic Ir and SrIrO_3_ perovskite phases (Figure 1h). The SAED pattern showing the arc‐like diffraction rings for the Ir nanoparticles indicates that these surface exsolved Ir nanoparticles are polycrystalline. As shown in energy dispersive spectroscopy (EDS) mapping (Figure 1i), there are a high concentration of Ir and a low concentration of Sr within the yellow circle, likely due to the segregation of Ir on SrIrO_3_ surface. These results collectively illustrate the successful construction of the Ir@SrIrO_3_ heterojunction by the partial exsolution approach.

**Figure 2:**
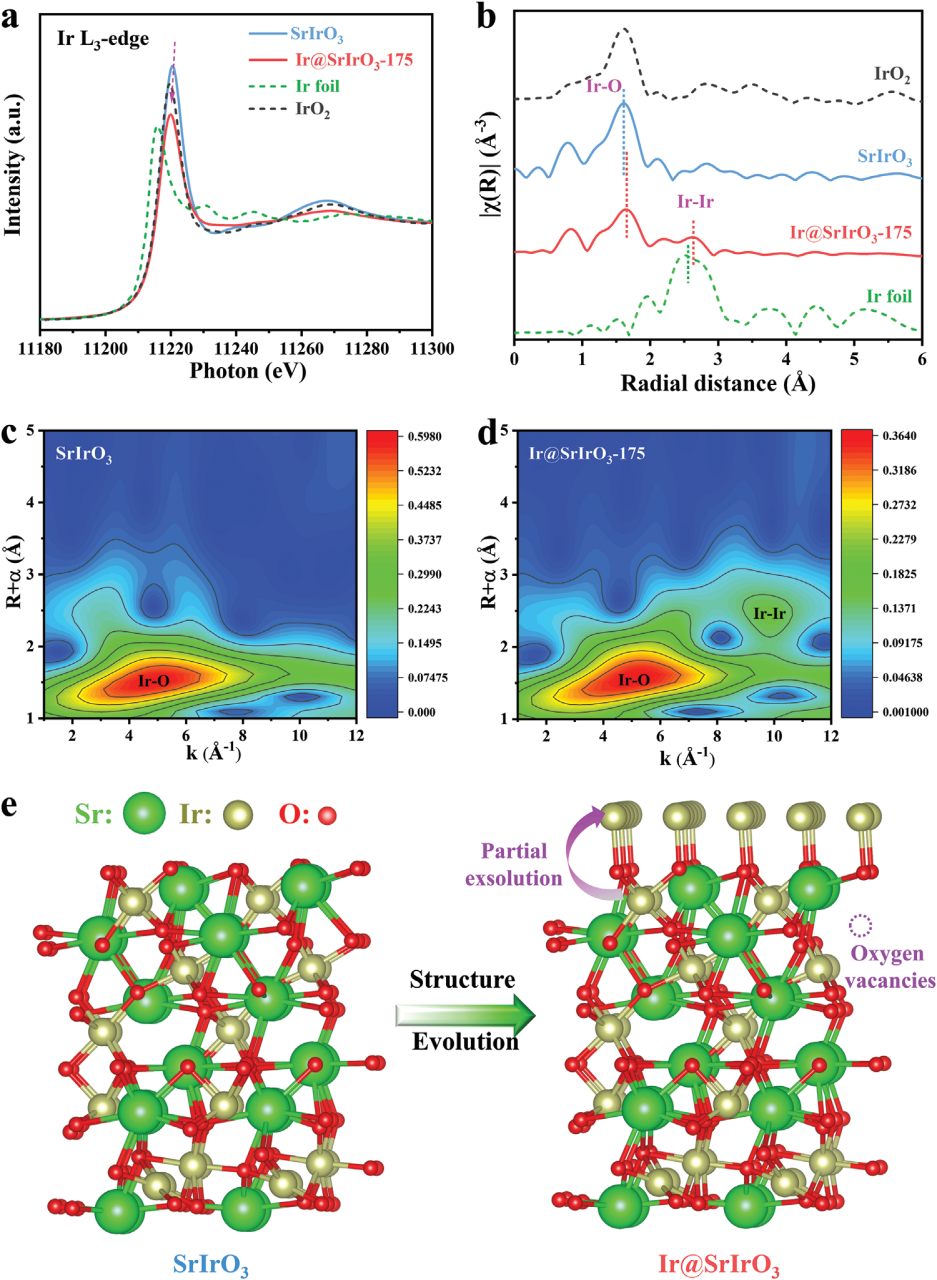
Structural analysis of electrocatalysts. a) Ir L_3_‐edge XANES and b) Ir L_3_‐edge EXAFS spectra of SrIrO_3_, Ir@SrIrO_3_‐175, IrO_2_, and Ir foil, Wavelet transform of Ir L_3_‐edge EXAFS data of c) SrIrO_3_ and d) Ir@SrIrO_3_‐175, e) Structural illustration of partial exsolution process.

Local electronic structures of SrIrO_3_ and Ir@SrIrO_3_‐175 electrocatalysts were probed via X‐ray absorption near‐edge structure spectroscopy (XANES) and extended X‐ray absorption fine structure spectroscopy (EXAFS) analysis. Ir foil (5d^7^6s^2^) and IrO_2_ (5d^5^6s^0^) served as the references. At Ir L_3_‐edge, the line intensity of Ir@SrIrO_3_‐175 is lower than that of SrIrO_3_, illustrative of a lower Ir valence in the Ir@SrIrO_3_‐175 heterojunction (**Figure** **2**a).^[^ ^25^ ^]^ This lower Ir valence further causes the increased Ir‐O interatomic distance of Ir@SrIrO_3_‐175 relative to SrIrO_3_ (Figure 2b).^[^ ^26^ ^]^ Compared to the pristine SrIrO_3_, the coordination of Ir‐O in the Ir@SrIrO_3_‐175 heterojunction is unsaturated, implying that rich oxygen vacancies are synchronously generated during the partial exsolution process (Table S2, Supporting Information). The creation of oxygen vacancies is also validated by the enlarged signal intensity (g around 2.002) in electron paramagnetic resonance (EPR) analysis (Figure S4, Supporting Information).^[^ ^27^ ^]^

Besides Ir─O bond, Ir─Ir bond is also confirmed in Ir@SrIrO_3_‐175 heterojunction,^[^ ^26^ ^]^ providing solid evidence of partial metallic Ir exsolution (Figure 2b). Remarkably, compared to individual SrIrO_3_ perovskite and metallic Ir, the shifts of Ir─O and Ir─Ir bonds in the Ir@SrIrO_3_‐175 heterojunction unveil a strong coupling interaction between Ir and SrIrO_3_. The wavelet transforms of SrIrO_3_ (Figure 2c) and Ir@SrIrO_3_‐175 (Figure 2d) further visualize the findings from EXAFS spectra. The fitting plots of the corresponding structures of SrIrO_3_ and Ir@SrIrO_3_‐175 in R space and k space are, respectively, provided in Figures S5 and S6 (Supporting Information). These XANES and EXAFS results evidentially demonstrate that the partial exsolution strategy creates Ir@SrIrO_3_ heterojunction endowed with the strong metal‐support interaction and the rich oxygen vacancies (Figure 2e).

### 2.2 Acidic OER and HER Activity

**Figure 3:**
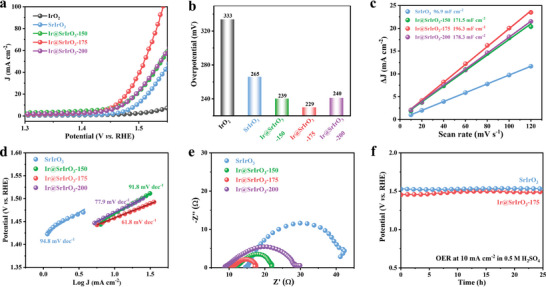
Electrochemical OER evaluation. a) LSV curves, b) Overpotentials, c) Double‐layer capacitances, d) Tafel plots, e) Nyquist curves of SrIrO_3_, Ir@SrIrO_3_‐150, Ir@SrIrO_3_‐175, Ir@SrIrO_3_‐200, and commercial IrO_2_ electrocatalysts, f) Chronoamperometric responses of SrIrO_3_ and Ir@SrIrO_3_‐175 electrocatalysts.

Using a standard three‐electrode system,^[^ ^28^ ^]^ the bifunctional activities of as‐prepared electrocatalysts towards OER and HER were extensively characterized in 0.5 M H_2_SO_4_ electrolyte. For comparing, commercial IrO_2_ and Pt/C were also evaluated for OER and HER, respectively. Analysis of the linear sweep voltammetry (LSV) curves (**Figure** **3**a) demonstrates that the pristine SrIrO_3_ electrocatalyst provides a higher OER activity in contrast to the commercial IrO_2_. Notably, constructing heterojunction further improves the OER activity. Among them, the Ir@SrIrO_3_‐175 electrocatalyst displays the best OER activity. Concretely, the Ir@SrIrO_3_‐175 electrocatalyst exhibits a lower overpotential of 229 mV at 10 mA cm^−2^ than those of SrIrO_3_ (265 mV), Ir@SrIrO_3_‐150 (239 mV), Ir@SrIrO_3_‐200 (240 mV), and benchmark IrO_2_ (333 mV) electrocatalysts (Figure 3b), which also surpasses the most of recently‐reported electrocatalysts in harsh acidic environment (Table S3, Supporting Information).^[^ ^10^, ^18^, ^19^, ^20^, ^21^, ^22^, ^23^, ^24^, ^25^, ^26^, ^27^, ^28^, ^29^ ^]^

Derived from CV curves (Figure S7, Supporting Information), the exsolved electrocatalysts demonstrate heightened C_dl_ values relative to the pristine SrIrO_3_ (Figure 3c), illustrative of more electrochemical active surface area (ECSA). Furthermore, the Ir@SrIrO_3_‐175 electrocatalyst delivers the largest ESCA‐normalized current density (Figure S8, Supporting Information), indicating the highest intrinsic OER activity among the studied electrocatalysts. It is thus concluded that the partial exsolution induces more active site and higher intrinsic activity. Additionally, the Ir@SrIrO_3_‐175 electrocatalyst exhibits a considerably lower Tafel slope of 61.8 mV dec^−1^ than 94.8 mV dec^−1^ for the pristine SrIrO_3_ (Figure 3d), denoting a faster OER kinetics. This trend in OER kinetics is also confirmed by electrochemical impedance spectra (EIS) analysis. Illustrated in Figure 3e, the Ir@SrIrO_3_‐175 electrocatalyst presents the lowest polarization resistance of ≈7 Ω compared to the pristine SrIrO_3_ (≈27 Ω), the Ir@SrIrO_3_‐150 (≈10 Ω), and the Ir@SrIrO_3_‐200 (≈21 Ω), implying the fastest charge transfer kinetics for OER.

To assess the stability of electrocatalyst, a chronopotentiometry test over Ir@SrIrO_3_‐175 electrocatalyst was performed under the acidic OER conditions. The enduring stabilities of the pristine SrIrO_3_ and the Ir@SrIrO3‐175 electrocatalysts during 25 h of continuous OER testing in acidic environment are evident in Figure 3f, where the potentials almost remain constant. As reported, the strong edge or face sharing [IrO_6_] configuration likely imparts high stability for a long‐term OER operation.^[^ ^30^ ^]^ Furthermore, by balancing the elemental dissolution in BaIr_1‐x_Mn_x_O_3_, a rigid surface is reconstructed, thereby exhibiting remarkably high OER durability.^[^ ^31^ ^]^ In the case of monoclinic SrIrO_3_, the surface‐reconstructed face‐sharing IrO_6_ octahedral subunits ensure high structural stability for acidic OER,^[^ ^10b^ ^]^ which might contribute to the exceptional durability of the Ir@SrIrO_3_‐175 electrocatalyst.

**Figure 4:**
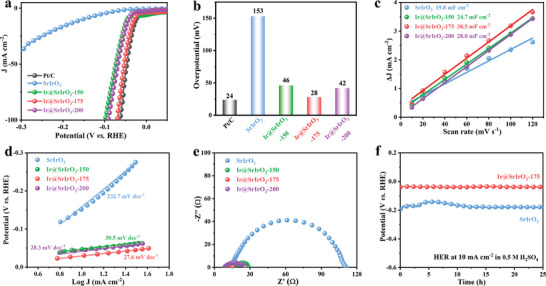
Electrochemical HER evaluation. a) LSV curves, b) Overpotentials, c) Double‐layer capacitances, d) Tafel plots, e) Nyquist curves of SrIrO_3_, Ir@SrIrO_3_‐150, Ir@SrIrO_3_‐175, Ir@SrIrO_3_‐200, and commercial Pt/C electrocatalysts, f) Chronoamperometric responses of SrIrO_3_ and Ir@SrIrO_3_‐175 electrocatalysts.

For HER, the LSV polarization curves of various electrocatalysts (SrIrO_3_, Ir@SrIrO_3_‐150, Ir@SrIrO_3_‐175, Ir@SrIrO_3_‐200, and commercial Pt/C are illustrated in **Figure** **4**a. The pristine SrIrO_3_ electrocatalyst displays an inferior HER activity. It is evident that the partial exsolution can activate the HER activity of SrIrO_3_ electrocatalyst. As shown in Figure 4b, the overpotentials for achieving a current density of ‐10 mA cm^−2^ are as follows: Pt/C (24 mV) < Ir@SrIrO_3_‐175 (28 mV) < Ir@SrIrO_3_‐200 (42 mV) < Ir@SrIrO_3_‐150 (46 mV) < SrIrO_3_ (153 mV). Of note, the HER activity of the optimal Ir@SrIrO_3_‐175 electrocatalyst is comparable to the benchmark Pt/C electrocatalyst and other reported electrocatalysts in acidic media (Table S3, Supporting Information).^[^ ^18^, ^19^, ^29^ ^]^ Moreover, the Ir@SrIrO_3_‐175 electrocatalyst demonstrates the largest ESCA (Figure 4c; Figure S9, Supporting Information) and the highest ECSA‐normalized current density (Figure S10, Supporting Information) towards acidic HER. This observed trend is further consistent with the Tafel (Figure 4d) and EIS (Figure 4e) results. The lowest Tafel slope together with the smallest polarization resistance of the Ir@SrIrO_3_‐175 electrocatalyst implies its most efficient kinetics for acidic HER. In addition, the pristine SrIrO_3_ and the Ir@SrIrO_3_‐175 electrocatalysts almost maintain high stability over 25 h for acidic HER (Figure 4f).

### 2.3 Acidic Overall Water Splitting Application

**Figure 5:**
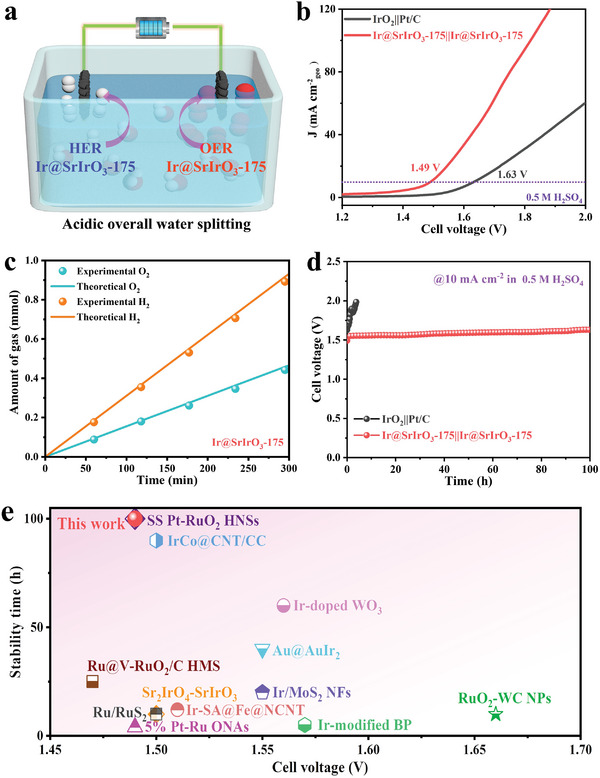
Acidic overall water splitting of Ir@SrIrO_3_‐175||Ir@SrIrO_3_‐175 based electrolyzer and benchmark IrO_2_||Pt/C based electrolyzer in 0.5 M H_2_SO_4_. a) Schematic diagram, b) Polarization plots, c) Amount of produced gas, d) Chronopotentiometry curves, and e) Comparison of water splitting performances with other bifunctional electrocatalysts.

Encouraged by the promising performances for both OER and HER, the Ir@SrIrO_3_‐175 electrocatalyst was further assessed as the bifunctional electrocatalyst for overall water splitting in a 0.5 M H_2_SO_4_ electrolyte (**Figure** **5**a). The Ir@SrIrO_3_‐175||Ir@SrIrO_3_‐175 based electrolyzer delivers an impressively low voltage of 1.49 V to achieve a current density of 10 mA cm^−2^, far beyond the benchmark IrO_2_||Pt/C based electrolyzer requiring 1.63 V for the same current density (Figure 5b). By comparing the theoretical and experimental volumes of oxygen and hydrogen products (Figure 5c), the Faradaic efficiencies of the Ir@SrIrO_3_‐175||Ir@SrIrO_3_‐175 electrolyzer approach 100%. A significant performance degradation is detected in the initial stage for the benchmark IrO_2_||Pt/C electrolyzer, whereas no discernible activity decay is observed on the Ir@SrIrO_3_‐175||Ir@SrIrO_3_‐175 electrolyzer for continuous 100 h operation (Figure 5d). Remarkably, the electrochemical performance and durability of the bifunctional Ir@SrIrO_3_‐175 electrolyzer outperforms most recently reported bifunctional electrocatalysts for acidic water splitting (Table S4, Supporting Information),^[^ ^18^, ^19^, ^29^ ^]^ demonstrating its potential commercial viability for acidic overall water electrolysis.

Considering the potential corrosion under the acidic OER conditions, the structure stability of Ir@SrIrO_3_‐175 electrocatalyst after long‐term OER test was investigated. Figure S11 (Supporting Information), depicts the persistence of metallic Ir and SrIrO_3_ perovskite phases in the Ir@SrIrO3‐175 electrocatalyst post‐OER. However, a reduction in their crystallinity implies the possible dissolution and corrosion of electrocatalyst during the acidic OER process. Furthermore, the TEM interplanar spacings of 0.279 nm and 0.227 nm are well indexed to SrIrO_3_ (1 3 0) and Ir (1 1 1) planes, as depicted in Figure S12 (Supporting Information). In addition, X‐ray photoelectron spectroscopy (XPS) measurements were performed for the Ir@SrIrO_3_‐175 electrocatalyst before and after OER. The XPS analysis of Ir displays Ir 4 f_7/2_ and Ir 4 f_5/2_ core‐level peaks (Figure S13, Supporting Information). Upon deconvolution, the valence state of Ir is dramatically increased, denoting surface oxidation of Ir during the OER electrocatalysis process.^[^ ^32^ ^]^

### 2.4 Study of Electrocatalytic Mechanism

**Figure 6:**
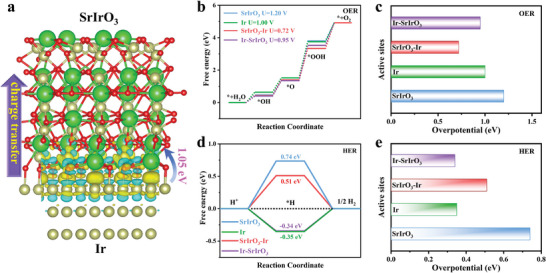
DFT calculations for OER and HER. a) Charge density difference of Ir‐SrIrO_3_ heterojunction, b) Free energy profiles of OER process, c) Corresponding OER theoretical overpotentials for SrIrO_3_,^[^ ^27a^ ^]^ Ir, SrIrO_3_‐Ir (SrIrO_3_ on top), and Ir‐SrIrO_3_ (Ir on top), d) Free energy profiles of HER process, e) Corresponding HER theoretical overpotentials for SrIrO_3_, Ir, SrIrO_3_‐Ir (SrIrO_3_ on top), and Ir‐SrIrO_3_ (Ir on top).

To elucidate the metal‐support interaction and the underlying bifunctional activity origin of the Ir@SrIrO_3_ heterojunction, Density functional theory (DFT) calculations on the interfacial electronic structure were performed. The heterojunction model was constructed by the experimentally exposed SrIrO_3_ (001)^[^ ^10b^ ^]^ and matched Ir (111) planes. The electron is enriched in interfacial region of Ir@SrIrO_3_ heterojunction, facilitating electron transfer for electrocatalytic reactions (**Figure** **6**a). Furthermore, it is calculated that the 1.05 eV electron are transferred from metallic Ir to SrIrO_3_ perovskite, rearranging the interfacial electron structure. This metal‐support interaction with electron redistribution is predicted to modulate the adsorption behavior of crucial intermediates.^[^ ^33^ ^]^ Previous reports indicate that the surface electron‐deficient precious metal sites can serve as active centers for various electrocatalytic reaction.^[^ ^18^, ^34^ ^]^

Moreover, we calculated the adsorption energy of OER and HER intermediates on SrIrO_3_ perovskite, metallic Ir, SrIrO_3_‐Ir (SrIrO_3_ on top), and Ir‐SrIrO_3_ (Ir on top) surfaces, respectively. It is well documented that the OER process follows the four proton‐coupled electron transfer steps. The relevant OER intermediates adsorbed on the surfaces are illustrated in Figures S14–S17 (Supporting Information), respectively. As shown in Gibbs free energy profiles for OER (Figure 6b), the conversion of *O to *OOH is the rate‐limiting step for SrIrO_3_ perovskite, metallic Ir, SrIrO_3_‐Ir, and Ir‐SrIrO_3_. It is evident that the SrIrO_3_‐Ir exhibits an OER energy barrier of 0.72 eV, superior to 1.20 eV on SrIrO_3_ perovskite, 1.00 eV on metallic Ir, and 0.95 eV on Ir‐SrIrO_3_ (Figure 6c), demonstrating the more favorable OER kinetics on the SrIrO_3_‐Ir. In the case of HER, the free energies of hydrogen adsorption on SrIrO_3_ perovskite, metallic Ir, SrIrO_3_‐Ir (SrIrO_3_ on top), and Ir‐SrIrO_3_ (Ir on top) surfaces were calculated (Figure S18, Supporting Information). One can see that the Ir‐SrIrO_3_ delivers the lowest HER energy barrier of 0.34 eV among them (Figure 6d,e). The above findings bolster the contention that SrIrO_3_‐Ir and Ir‐SrIrO_3_ possess high theoretical activities toward OER and HER, respectively, conferring high intrinsic bifunctionality to the Ir@SrIrO_3_ heterojunction for overall water splitting.

## 3 Conclusion

In summary, we have demonstrated the successful synthesis of novel Ir@SrIrO_3_ heterojunction by a top‐down partial exsolution strategy, yielding efficient and robust acidic overall water splitting. After the partial exsolution, the bifunctional activity of Ir@SrIrO_3_‐175 electrocatalyst prepared via annealing SrIrO_3_ in 5% H_2_ atmosphere at 175 °C is remarkably enhanced, delivering low overpotentials of 229 mV at 10 mA cm^−2^ for OER and 28 mV at 10 mA cm^−2^ for HER. More importantly, its application in acidic overall water splitting shows high efficiency of 1.49 V for 10 mA cm^−2^ and long‐term stability over 100 h, which outperforms the benchmark IrO_2_||Pt/C and is comparable to other excellent bifunctional electrocatalysts. DFT calculations further substantiate the improved bifunctional activity, attributed to the regulated interfacial electronic structure and favourable adsorption energy of intermediates. These findings underscore the potential of metal‐perovskite heterojunctions in synergistic electrocatalysis for sustainable energy conversion applications.

## 4 Experimental Section

### Electrocatalyst Synthesis

The synthesis of SrIrO_3_ perovskite nanosheets was carried out using a typical sol‐gel method. Initially, a solution (A) was prepared by dissolving 560 mg of Sr(NO_3_)_2_ and 560 mg of citric acid in 20 mL of deionized water. Concurrently, a solution (B) was formed by adding 380 mg of H_2_IrCl_6_·xH_2_O in 8 mL of ethylene glycol. Subsequently, the solution A was gradually introduced into the solution B. KOH solution was also added until the pH value of resulting mixture reaching ≈10. This obtained solution was heated at 150 °C for 12 h to yield a brown dry gel, following by calcination in air at 700 °C for 6 h. The preliminary black powder was further acid‐washed with 1 mol L^−1^ HCl for 8 h to remove residual SrCO_3_ impurities, yielding the target SrIrO_3_ electrocatalyst. For the preparation of Ir@SrIrO_3_ heterojunction, SrIrO_3_ powder was evenly spread in a porcelain boat, which was subjected to annealing in a tubular furnace under an 5% H_2_‐95% N_2_ atmosphere at various temperatures (150 °C, 175 °C, and 200 °C) for 10 min. For simplicity, the electrocatalysts treated at 150 °C, 175 °C, and 200 °C were respectively denoted as Ir@SrIrO_3_‐150, Ir@SrIrO_3_‐175, and Ir@SrIrO_3_‐200.

### Structural Characterization

X‐ray diffraction (XRD) using a Bruker D8 Advance with Cu K𝛼 radiation was employed to identify the phases of electrocatalysts. The microstructures of electrocatalysts were examined by scanning electron microscopy (SEM) and high‐resolution transmission electron microscopy (HRTEM) on Hitachi SU‐8010 and Titan G260‐300 instruments, respectively. High angle annular dark field‐scanning transmission electron microscopy (HAADF‐STEM) was carried out by an aberration‐corrected Hitachi 2700D microscope. X‐ray absorption spectroscopy (XAS) at Ir L_3_‐edge was performed at the BL14W1 beamline of the Shanghai Synchrotron Radiation Facility, with Ir foil and IrO_2_ serving as reference standards. Analysis of the XAS spectra was conducted via the ATHENA program. Surface electronic structures of the electrocatalysts were probed using X‐ray photoelectron spectroscopy (XPS) with monochromatic Al K𝛼 radiation on a K‐ALPHA instrument. Electron paramagnetic resonance (EPR) measurements were carried out on a Bruker‐A300 spectrometer to assess the oxygen vacancy concentration in electrocatalysts.

### Electrochemical Measurement

Electrochemical characterization of the synthesized electrocatalysts was conducted using a CHI‐760E electrochemical workstation in a standard three‐electrode system. A 0.5 M H_2_SO_4_ solution served as the acidic electrolyte. The setup included a saturated calomel reference electrode, a carbon rod counter electrode, and a working electrode comprising the electrocatalyst on a glassy carbon electrode with Nafion binder (loading of 0.23 mg cm^−2^). The activity and durability for OER and HER were assessed through linear sweep voltammetry (LSV) at 10 mV s^−1^, cyclic voltammetry (CV), electrochemical impedance spectroscopy (EIS) from 100 kHz to 0.1 Hz, and chronopotentiometry. Measured potentials were referenced to RHE and corrected for potential loss from electrolyte resistance with an 85% iR‐drop compensation.

Acidic water splitting performance was evaluated in a two‐electrode setup in 0.5 M H_2_SO_4_ electrolyte. The bifunctional Ir@SrIrO_3_‐175 electrocatalyst loaded on carbon cloth (1 mg cm^−2^) was used as both the anode and cathode of electrolyzer. For comparison, an electrolyzer with benchmark IrO_2_||Pt/C electrodes at the identical loading was also prepared. Polarization curves and chronopotentiometric measurements were recorded without iR compensation. Faraday efficiency was determined by measuring the volumes of O_2_ and H_2_ during water splitting.

### Theoretical Calculation

Density functional theory (DFT) calculations,^[^ ^35^ ^]^ utilizing the generalized gradient approximation (GGA) with the Perdew‐Burke‐Ernzerhof (PBE) exchange‐correlation functional,^[^ ^36^ ^]^ were implemented using the VASP software to explore the electronic properties of electrocatalysts. The long‐range van der Waals interactions were accounted for using the DFT‐D3 approach. The plane wave cutoff energy was set at 500 eV, and the energy convergence criterion for the iterative solution of the Kohn‐Sham equation was established at 10^−5^ eV. To prevent artificial interactions between periodic images, a vacuum layer of 15 Å was introduced perpendicular to the material surface. The experimentally exposed (001) plane of monoclinic SrIrO_3_ perovskite was applied for model specification. The heterojunction derived from the segments of SrIrO_3_ (001) and matched Ir (111), was constructed to investigate the synergy catalysis between SrIrO_3_ perovskite and metal Ir. Structural relaxation was continued until the residual forces on atoms reduced to less than 0.02 eV Å^−1^. Surface Ir on SrIrO_3_, Ir, SrIrO_3_‐Ir (SrIrO_3_ on top), and Ir‐SrIrO_3_ (Ir on top) were regarded as electrochemically active sites. For OER reaction pathway, the adsorption free energies of OH*, O* and OOH* were calculated. Likewise, the adsorption free energies of H* were calculated for HER.^[^ ^18^ ^]^

## Conflict of Interest

The authors declare no conflict of interest.

## Supporting information

Supporting Information

## Data Availability

The data that support the findings of this study are available in the supplementary material of this article.
