# Supplementary material for: Partial Exsolution Enables Superior Bifunctionality of Ir@SrIrO3 for Acidic Overall Water Splitting
Source: Adv Sci (Weinh). 2024 Apr 2;11(24):2309750. doi: 10.1002/advs.202309750 (PMC11199977; doi:10.1002/advs.202309750)
Supplement: Supplementary file 1 — Supporting Information [file ADVS-11-2309750-s001.pdf]

ADVANCED SCIENCE
Open Access

Supporting Information

for *Adv. Sci.*, DOI 10.1002/advs.202309750

Partial Exsolution Enables Superior Bifunctionality of Ir@$SrIrO_3$ for Acidic Overall Water Splitting

Ling Zhao, Zetian Tao, Maosheng You, Huangwei Xiao, Sijiao Wang, Wenjia Ma, Yonglong Huang, Beibei He and Qi Chen**

Supporting information

Partial Exsolution Enables Superior Bifunctionality of Ir@$SrIrO_3$ for Acidic Overall Water Splitting

Ling Zhao, Zetian Tao, Maosheng You, Huangwei Xiao, Sijiao Wang, Wenjia Ma, Yonglong Huang, Beibei He, Qi Chen.**

Prof L. Zhao, Prof. Q. Chen, School of Marine Science and Engineering, Hainan University, Haikou 570228, PR China. Email: chenqi@hainanu.edu.cn.

Prof. L. Zhao, M.S. You, H.W. Xiao, S.J. Wang, W.J. Ma, Y.L. Huang, Prof. B.B. He, Faculty of Materials Science and Chemistry, China University of Geosciences, Wuhan, 430074, China. Email: babyfly@mail.ustc.edu.cn.

Prof. Z.T. Tao, School of Resources, Environment and Safety Engineering, University of South China, Hengyang, Hunan Province, 421001, China.

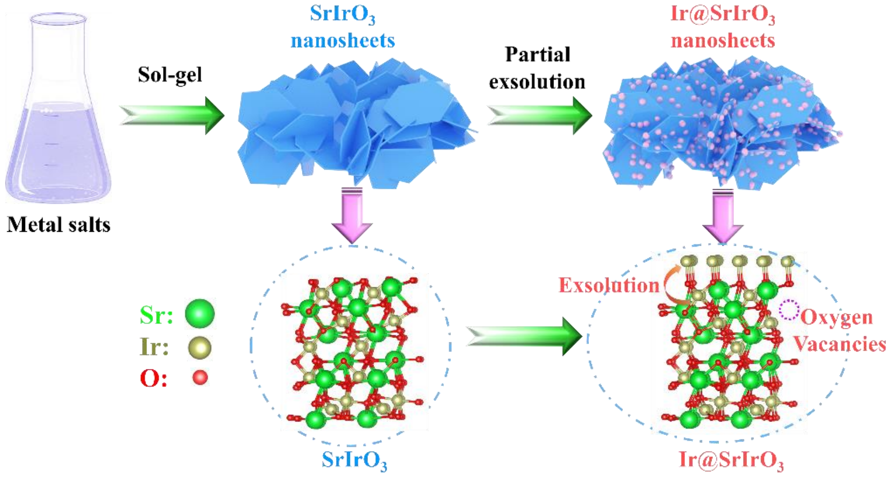

Figure S1. Schematic diagram of the synthesis of partially exsolved $Ir@SrIrO_3$ heterojunction.

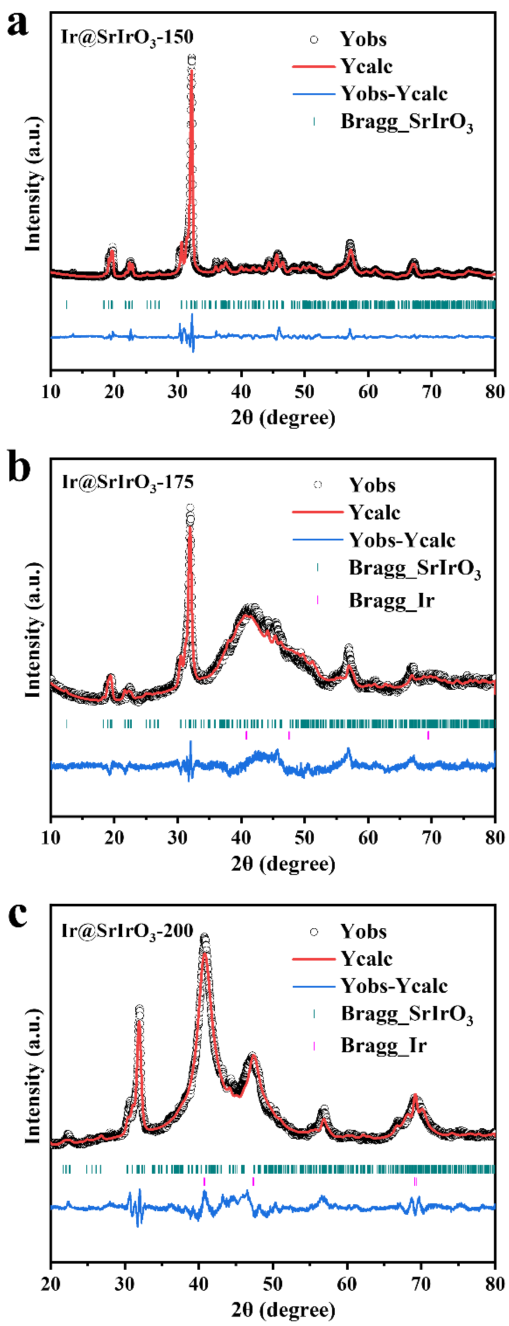

Figure S2. Rietveld refinements of (a) Ir@$SrIrO_3$-150, (b) Ir@$SrIrO_3$-175, and (c) Ir@$SrIrO_3$-200 electrocatalysts.

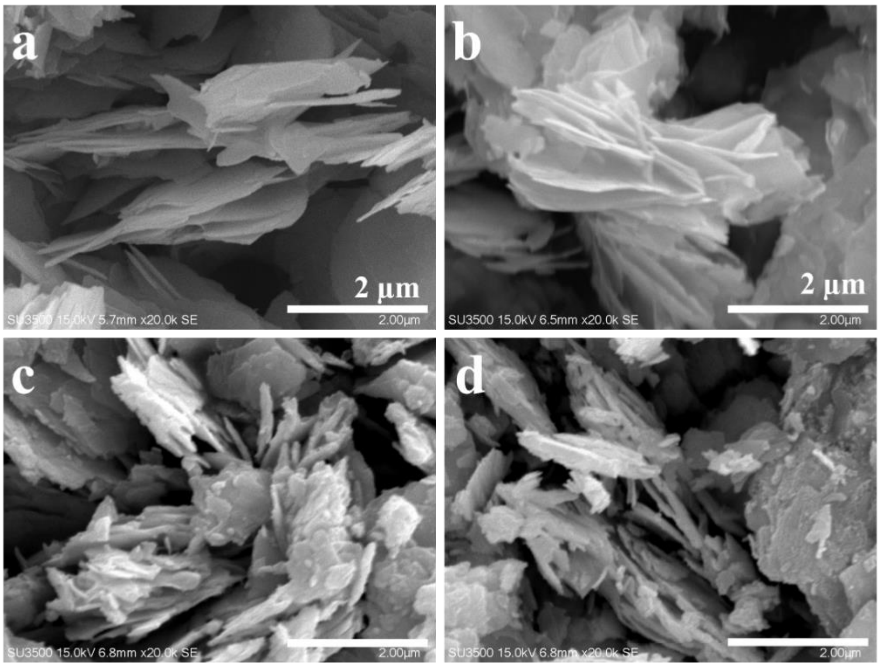

Figure S3. FESEM images of (a) $SrIrO_3$, (b) Ir@$SrIrO_3$-150, (c) Ir@$SrIrO_3$-175, and (d) Ir@$SrIrO_3$-200 electrocatalysts.

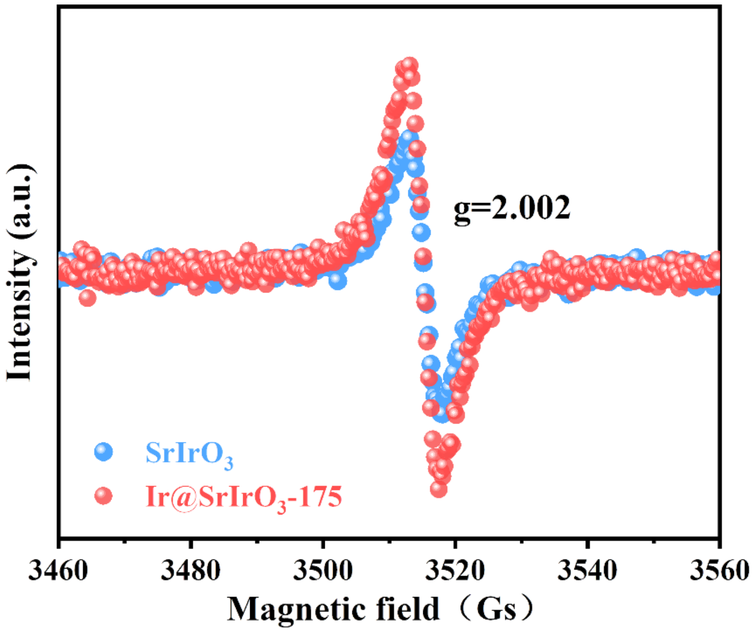

Figure S4. EPR spectra of $SrIrO_3$ and Ir@$SrIrO_3$-175 electrocatalysts.

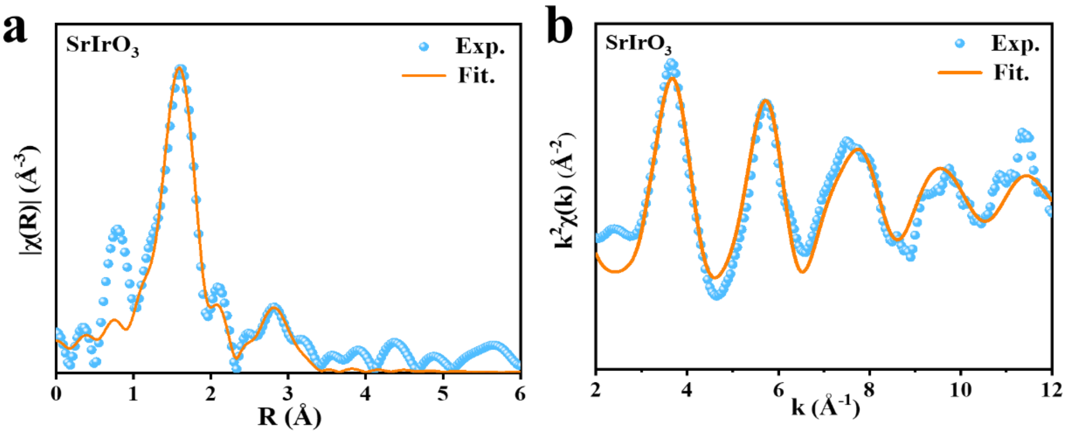

Figure S5. Fitting result of corresponding structure of $SrIrO_3$ in a) R space, b) k space.

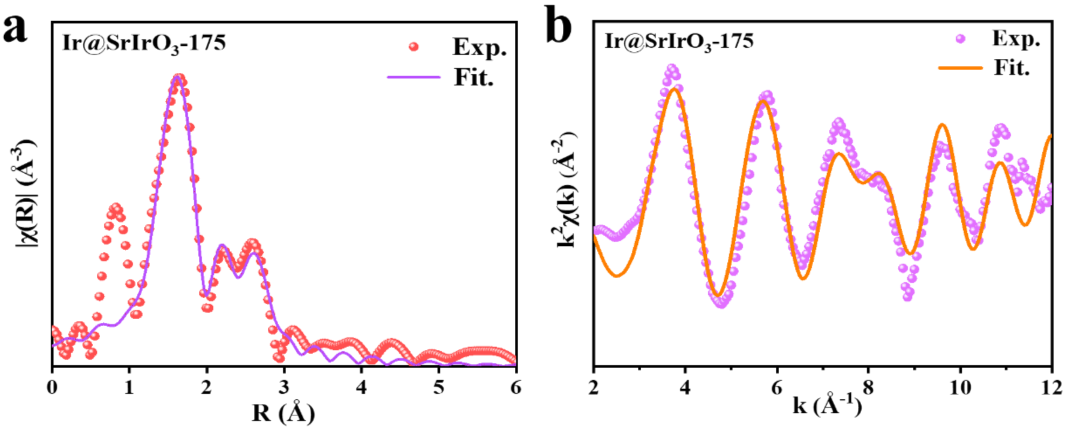

Figure S6. Fitting result of corresponding structure of Ir@$SrIrO_3$-175 in a) R space, b) k space.

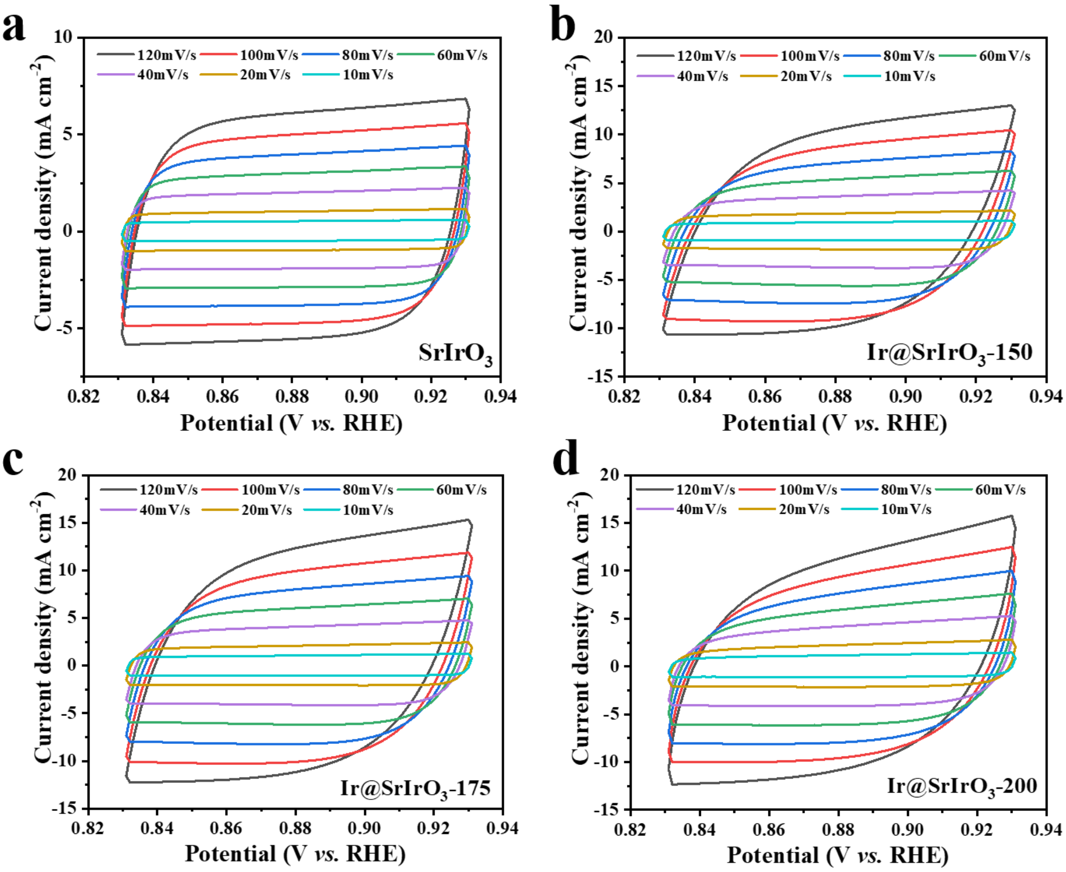

Figure S7. Cyclic voltammograms of (a) $SrIrO_3$, (b) Ir@$SrIrO_3$-150, (c) Ir@$SrIrO_3$-175, and (d) Ir@$SrIrO_3$-200 electrocatalysts rating from 10 to 120 mV in 0.5 M H_2SO_4 for OER.

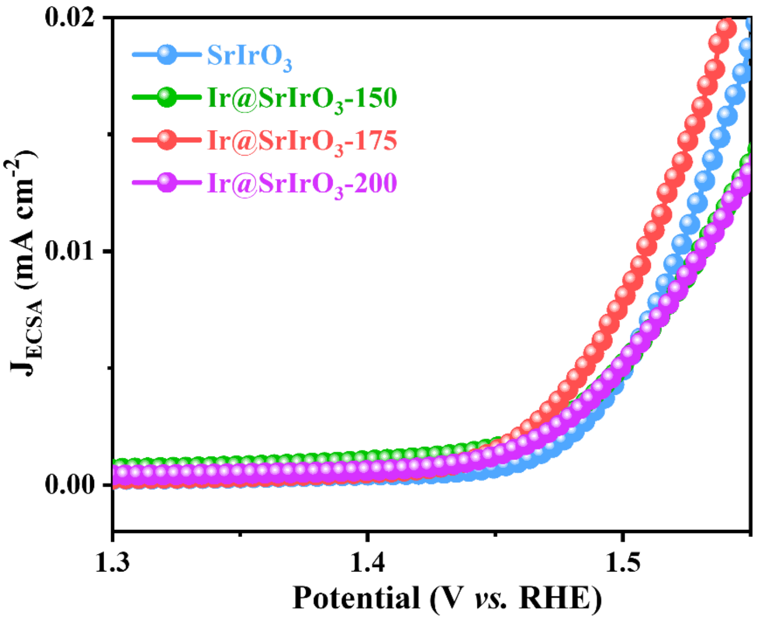

Figure S8. Polarization curves with normalized by ECSA of $SrIrO_3$, Ir@$SrIrO_3$-150, Ir@$SrIrO_3$-175, and Ir@$SrIrO_3$-200 electrocatalysts for OER.

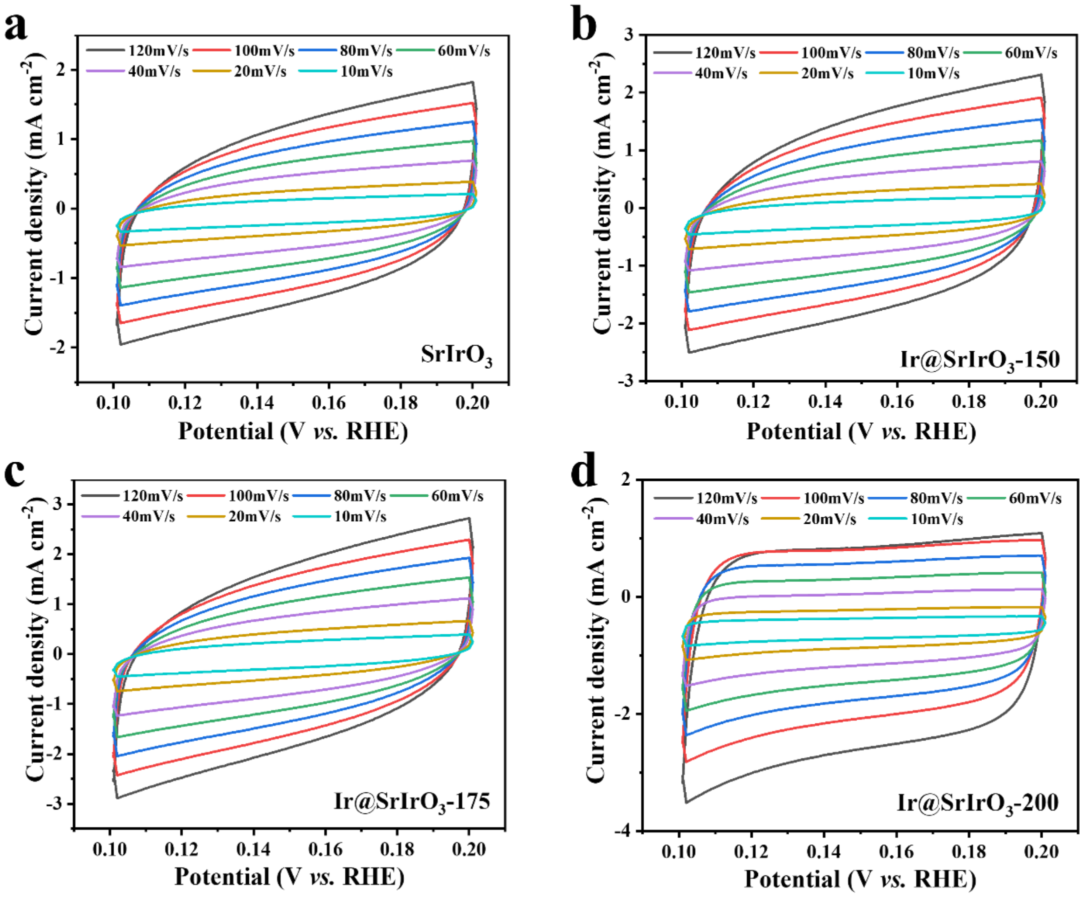

Figure S9. Cyclic voltammograms of (a) $SrIrO_3$, (b) Ir@$SrIrO_3$-150, (c) Ir@$SrIrO_3$-175, and (d) Ir@$SrIrO_3$-200 electrocatalysts rating from 10 to 120 mV in 0.5 M H_2SO_4 for HER.

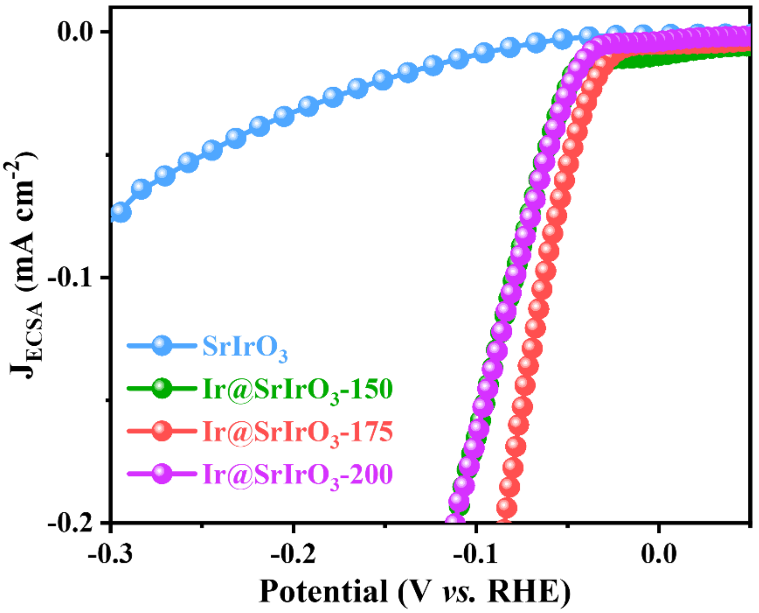

Figure S10. Polarization curves with normalized by ECSA of $SrIrO_3$, $Ir@SrIrO_3$-150, $Ir@SrIrO_3$-175, and $Ir@SrIrO_3$-200 electrocatalysts for HER.

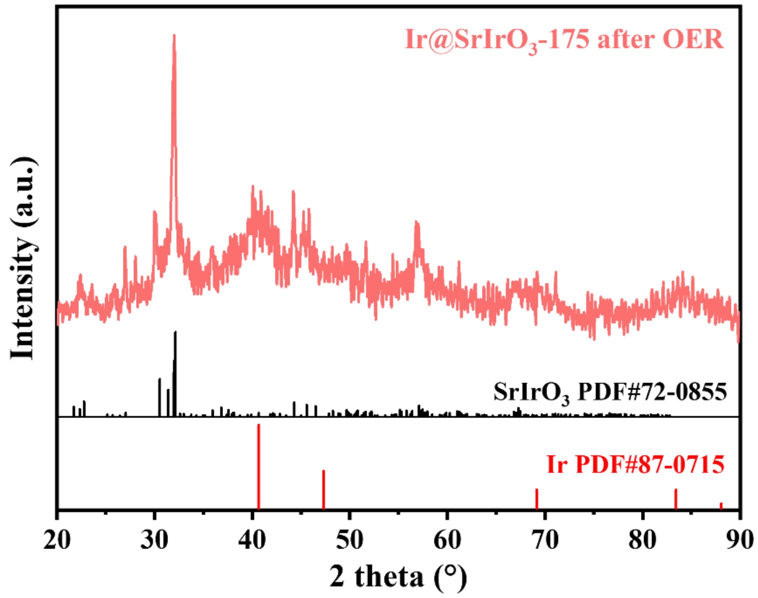

Figure S11. XRD pattern of $Ir@SrIrO_3$-175 electrocatalyst after long-term acidic OER operation.

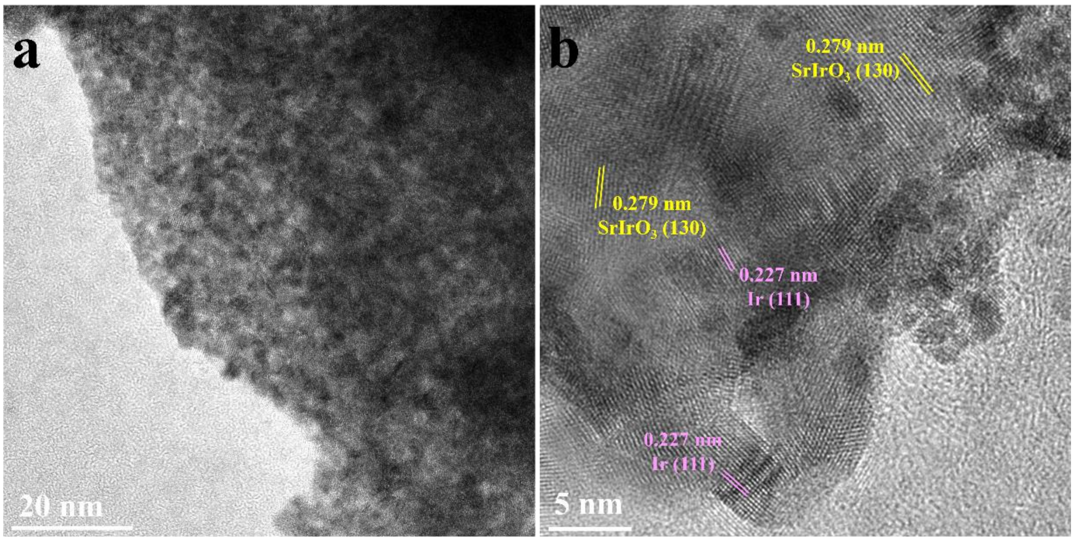

Figure S12. TEM images of Ir@$SrIrO_3$-175 electrocatalyst after long-term acidic OER operation.

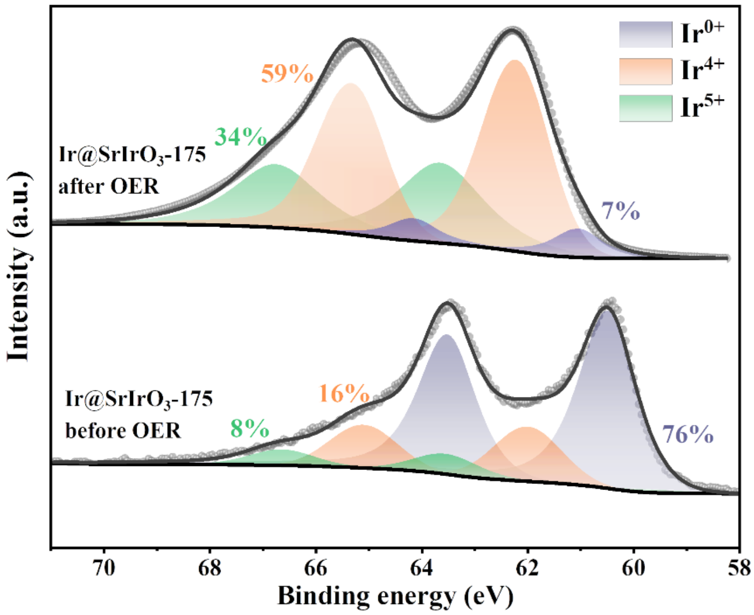

Figure S13. XPS Ir 4f spectra of Ir@$SrIrO_3$-175 electrocatalyst before and after long-term acidic OER operation.

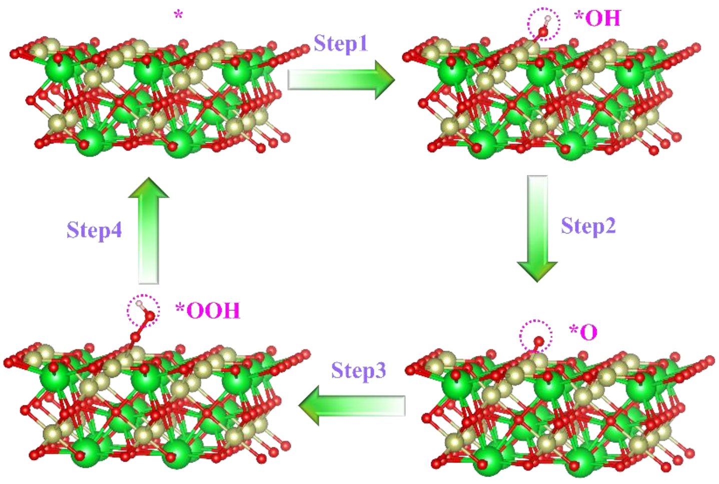

Figure S14. Theoretical structural models of OER intermediates adsorbed on $SrIrO_3$. (Ir atom: golden, Sr atom: green, O atom: red, H atom: pink).

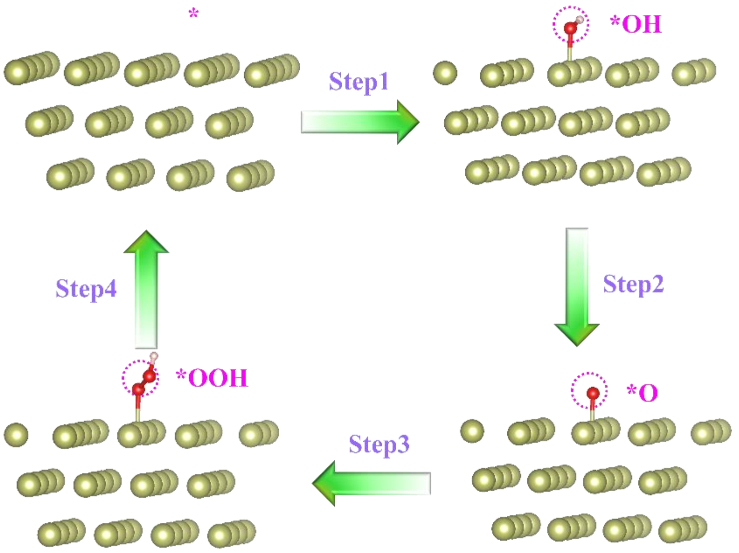

Figure S15. Theoretical structural models of OER intermediates adsorbed on Ir. (Ir atom: golden, O atom: red, H atom: pink).

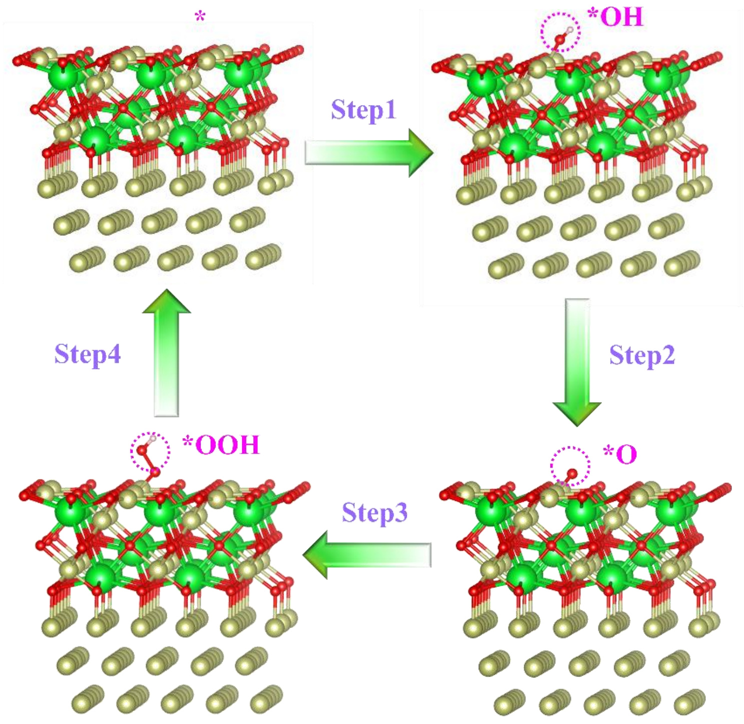

Figure S16. Theoretical structural models of OER intermediates adsorbed on $SrIrO_3$-Ir ($SrIrO_3$ on top). (Ir atom: golden, Sr atom: green, O atom: red, H atom: pink).

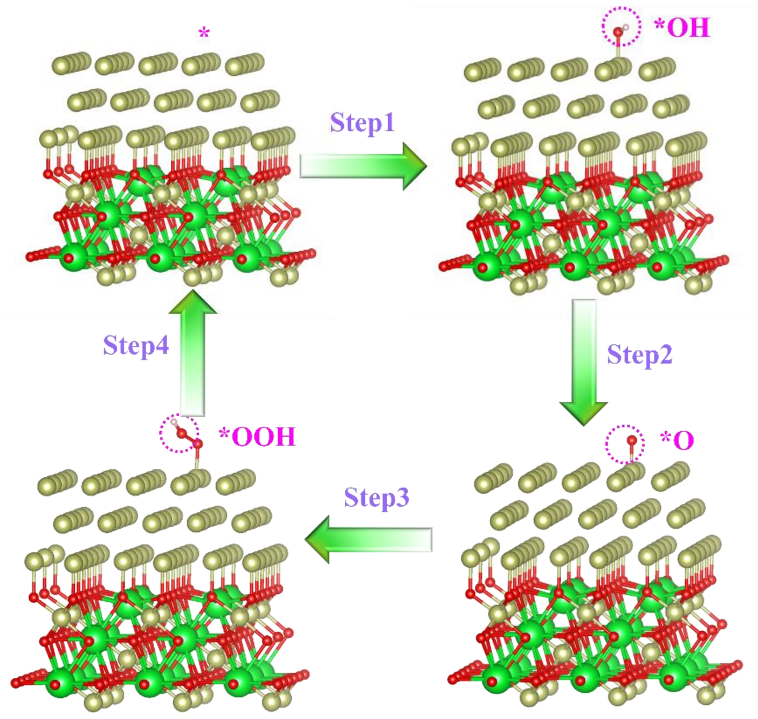

Figure S17. Theoretical structural models of OER intermediates adsorbed on Ir-$SrIrO_3$ (Ir on top). (Ir atom: golden, Sr atom: green, O atom: red, H atom: pink).

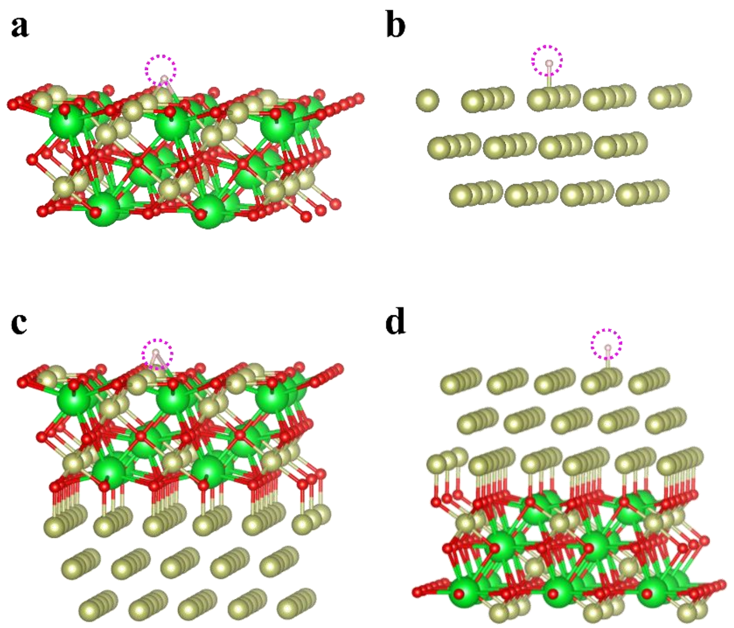

Figure S18. Theoretical structural models of HER intermediate (H*) adsorbed on (a) $SrIrO_3$, (b) Ir, (c) $SrIrO_3$-Ir ($SrIrO_3$ on top), and (d) Ir-$SrIrO_3$ (Ir on top) surfaces. (Ir atom: golden, Sr atom: green, O atom: red, H atom: pink).

Table S1. Crystallographic information for Ir@$SrIrO_3$-150, Ir@$SrIrO_3$-175, and Ir@$SrIrO_3$-200 electrocatalysts according to Rietveld refinements.

Materials	Ir@$SrIrO_3$-150	Ir@$SrIrO_3$-175		Ir@$SrIrO_3$-200	
Phase structure	C2/c	C2/c	Fm-3m	C2/c	Fm-3m
a (Å)	5.59338	5.62494	3.83004	5.61226	3.84055
b (Å)	9.61959	9.67539	3.83004	9.73434	3.84055
c (Å)	14.18057	14.23297	3.83004	14.34772	3.84055
α (°)	90	90	90	90	90
β (°)	93.2162	93.09709	90	92.86150	90
γ (°)	90	90	90	90	90
V ($Å^3$)	761.799	773.476	56.184	782.862	56.647
Fract (%)	100	29.56	70.44	15.88	84.12
R_p	8.23	11.2		12.2	
R_{wp}	10.8	13.1		13.7	
χ^2	4	4.64		6.48	

Table S2. EXAFS Fitting parameters of $SrIrO_3$ and Ir@$SrIrO_3$-175 electrocatalysts.

Catalysts	Path	C.N.	R (Å)	$\sigma^2\times10^3$ ($Å^2$)	R factor
$SrIrO_3$	Ir-O	6.1±0.7	1.98±0.01	4.0±1	0.017

Ir-$SrIrO_3$	Ir-O	3.2±0.6	1.99±0.01	2.8±2	0.016

S_0^2 fixed at 0.9 for Ir

Table S3. Comparation of the overpotentials of the OER and HER over Ir@$SrIrO_3$-175 with the reported electrocatalysts at 10 mA cm^{-2} in acid media.

Catalyst	Electrolyte	OER (mV) 10 mA cm^{-2}	HER (mV) 10 mA cm^{-2}	Ref
Ir@$SrIrO_3$-175	0.5M H_2SO_4	229	28	This work
Sr_2IrO_4-$SrIrO_3$	0.5M H_2SO_4	263	18	Chem. Eng. J. 2021, 419, 129604[S1]
Ir/MoS_2 NFs	0.5M H_2SO_4	270	35	J. Energy Chem. 2023, 87, 144[S2]
Au@$AuIr_2$	0.5M H_2SO_4	261	29	J. Am. Chem. Soc. 2021, 143, 4639[S3]
Ir-modified BP	0.5M H_2SO_4	290	26	Adv. Mater. 2021, 33, 2104638[S4]
IrCo@CNT/CC	0.5M H_2SO_4	241	26	ACS Catal. 2023, 13, 10672[S5]
Ir-SA@Fe@NCNT	0.5M H_2SO_4	250	26	Nano Lett. 2020, 20, 2120[S6]
Ir-doped WO_3	0.5M H_2SO_4	258	36	Small 2021, 17, 2102078[S7]
Ru@V-RuO_2/C HMS	0.5M H_2SO_4	176	46	Adv. Mater.2023, 35, 2206351[S8]
Ru/RuS_2	0.5M H_2SO_4	201	45	Angew. Chem. Int. Ed. 2021, 60, 12328[S9]
SS Pt-RuO_2 HNSs	0.5M H_2SO_4	228	26	Sci. Adv. 2022, 8, eabl9271[S10]
5% Pt-Ru ONAs	0.5M H_2SO_4	227	31	Chinese J. Catal. 2022, 43, 1493[S11]
RuO_2-WC NPs	0.5M H_2SO_4	347	58	Angew. Chem. Int. Ed. 2022, 61, e202202519[S12]
IrO_x/$SrIrO_3$	0.5M H_2SO_4	270-290	-	Science 2016, 353, 1011[S13]
6H-$SrIrO_3$	0.5M H_2SO_4	255	-	Nat. Commun. 2018, 9, 5236[S14]

Table S4. Comparation of electrochemical performance of overall acidic water splitting using Ir@$SrIrO_3$-175 and other excellent bifunctional electrocatalysts in acid media.

Catalyst	Electrolyte	Voltage (V) @10 mA cm^{-2}	Stability (h)	Ref
Ir@$SrIrO_3$-175	0.5M H_2SO_4	1.49	100	This work
Sr_2IrO_4-$SrIrO_3$	0.5M H_2SO_4	1.50	10	Chem. Eng. J. 2021, 419, 129604[S1]
Ir/MoS_2 NFs	0.5M H_2SO_4	1.55	20	J. Energy Chem. 2023, 87, 144[S2]
Au@$AuIr_2$	0.5M H_2SO_4	1.55	40	J. Am. Chem. Soc. 2021, 143, 4639[S3]
Ir-modified BP	0.5M H_2SO_4	1.57	5	Adv. Mater. 2021, 33, 2104638[S4]
IrCo@CNT/CC	0.5M H_2SO_4	1.50	90	ACS Catal. 2023, 13, 10672[S5]
Ir-SA@Fe@NCNT	0.5M H_2SO_4	1.51	12	Nano Lett. 2020, 20, 2120[S6]
Ir-doped WO_3	0.5M H_2SO_4	1.56	60	Small 2021, 17, 2102078[S7]
Ru@V-RuO_2/C HMS	0.5M H_2SO_4	1.47	25	Adv. Mater.2023, 35, 2206351[S8]
Ru/RuS_2	0.5M H_2SO_4	1.50	10	Angew. Chem. Int. Ed. 2021, 60, 12328[S9]
SS Pt-RuO_2 HNSs	0.5M H_2SO_4	1.49	100	Sci. Adv. 2022, 8, eabl9271[S10]
5% Pt-Ru ONAs	0.5M H_2SO_4	1.49	4	Chinese J. Catal. 2022, 43, 1493[S11]
RuO_2-WC NPs	0.5M H_2SO_4	1.66	10	Angew. Chem. Int. Ed. 2022, 61, e202202519[S12]

References:

[S1] Zhang, L.; Jang, H.; Li, Z.; Liu, H.; Kim, M. G.; Liu, X.; Cho, J. P. $SrIrO_3$ modified with laminar Sr_2IrO_4 as a robust bifunctional electrocatalyst for overall water splitting in acidic media. *Chem. Eng. J.* **2021**, *419* (129604). DOI: 10.1016/j.cej.2021.129604.

[S2] Wang, C.; Yu, L.; Yang, F.; Feng, L. MoS_2 nanoflowers coupled with ultrafine Ir nanoparticles for efficient acid overall water splitting reaction *J. Energy Chem.* **2023**, *87* (144). DOI: 10.1016/j.jechem.2023.08.017.

[S3] Wang, H.; Chen, Z.; Wu, D.; Cao, M.; Sun, F.; Zhang, H.; You, H.; Zhuang, W.;

Cao, R. Significantly enhanced overall water splitting performance by partial oxidation of Ir through Au modification in core–shell alloy structure. *J. Am. Chem. Soc.* **2021**, *143* (4639). DOI: 10.1021/jacs.0c12740.
[S4] Mei, J.; He, T.; Bai, J.; Qi, D.; Du, A.; Liao, T.; Ayoko, G. A.; Yamauchi, Y.; Sun, L.; Sun, Z. Surface-dependent intermediate adsorption modulation on iridium-modified black phosphorus electrocatalysts for efficient pH-universal water splitting. *Adv. Mater.* **2021**, *33* (2104638). DOI: 10.1002/adma.202104638.
[S5] Wang, X.; Qin, Z.; Qian, J.; Chen, L.; Shen, K. IrCo nanoparticles encapsulated with carbon nanotubes for efficient and stable acidic water splitting. *ACS Catal.* **2023**, *13* (10672). DOI: 10.1021/acscatal.3c02887.
[S6] Luo, F.; Hu, H.; Zhao, X.; Yang, Z.; Zhang, Q.; Xu, J.; Kaneko, T.; Yoshida, Y.; Zhu, C.; Cai, W. Robust and stable acidic overall water splitting on Ir single atoms. *Nano Lett.* **2020**, *20* (2120). DOI: 10.1021/acs.nanolett.0c00127.
[S7] Li, P.; Duan, X.; Kuang, Y.; Sun, X. Iridium in Tungsten Trioxide Matrix as an Efficient Bi-Functional Electrocatalyst for Overall Water Splitting in Acidic Media. *Small* **2021**, *17* (2102078). DOI: 10.1002/smll.202102078.
[S8] Li, Y.; Wang, W.; Cheng, M.; Feng, Y.; Han, X.; Qian, Q.; Zhu, Y.; Zhang, G. Arming Ru with Oxygen-Vacancy-Enriched RuO_2 Sub-Nanometer Skin Activates Superior Bifunctionality for pH-Universal Overall Water Splitting *Adv. Mater.* **2023**, *35* (e2206351). DOI: 10.1002/adma.202206351.
[S9] Zhu, J.; Guo, Y.; Liu, F.; Xu, H.; Gong, L.; Shi, W.; Chen, D.; Wang, P.; Yang, Y.; Zhang, C.; Wu, J.; Luo, J.; Mu, S. Regulative electronic states around ruthenium/ruthenium disulphide heterointerfaces for efficient water splitting in acidic media. *Angew. Chem. Int. Ed.* **2021**, *60* (12328). DOI: 10.1002/anie.202101539.
[S10] Wang, J.; Yang, H.; Li, F.; Li, L.; Wu, J.; Liu, S.; Cheng, T.; Xu, Y.; Shao, Q.; Huang, X. Single-site Pt-doped RuO_2 hollow nanospheres with interstitial C for high-performance acidic overall water splitting. *Sci. Adv.* **2022**, *8*, (eabl9271). DOI: 10.1126/sciadv.abl9271.
[S11] Yao, Q.; Le, J.; Yang, S.; Cheng, J.; Shao, Q.; Huang, X. A trace of Pt can significantly boost RuO_2 for acidic water splitting. *Chinese J. Catal.* **2022**, *43* (1493). DOI: 10.1016/S1872-2067(21)63952-9.
[S12] Sun, S.; Jiang, H.; Chen, Z.; Chen, Q.; Ma, M.; Zhen, L.; Song, B.; Xu, C. Bifunctional WC-Supported RuO_2 Nanoparticles for Robust Water Splitting in Acidic Media. *Angew. Chem. Int. Ed.* **2022**, 61, (e202202519). DOI: 10.1002/ange.202202519.
[S13] Seitz, L. C.; Dickens, C. F.; Nishio, K.; Hikita, Y.; Montoya, J.; Doyle, A.; Kirk, C.; Vojvodic, A.; Hwang, H. Y.; Norskov, J. K.; Jaramillo, T. F. A highly active and stable $IrO_x/SrIrO_3$ catalyst for the oxygen evolution reaction. *Science* **2016**, *353* (1011). DOI: 10.1126/science.aaf5050.
[S14] Yang, L.; Yu, G.; Ai, X.; Yan, W.; Duan, H.; Chen, W.; Li, X.; Wang, T.; Zhang, C.; Huang, X.; Chen, J.; Zou, X. Efficient oxygen evolution electrocatalysis in acid by a perovskite with face-sharing IrO_6 octahedral dimers *Nat. Commun.* **2018**, *9* (5236). DOI: 10.1038/s41467-018-07678-w.
